# The association between climate, geography and respiratory syncitial virus hospitalizations among children in Ontario, Canada: a population-based study

**DOI:** 10.1186/s12879-020-4882-6

**Published:** 2020-02-19

**Authors:** Dhenuka Radhakrishnan, Alexandra Ouedraogo, Salimah Z. Shariff, J. Dayre McNally, Eric I. Benchimol, Kristin K. Clemens

**Affiliations:** 1 Children’s Hospital of Eastern Ontario Research Institute, 401 Smyth Road, Ottawa, ON K1H 8L1 Canada; 2 Department of Pediatrics, University of Ottawa, Ottawa, ON Canada; 3 ICES, Toronto, Ontario Canada; 4 School of Epidemiology and Public Health, University of Ottawa, Ottawa, Canada; 5 Department of Medicine & Department of Epidemiology and Biostatistics, Western University, London, ON Canada; 6 St. Joseph’s Health Care London, PO Box 5777, STN B, London, ON N6A 4V2 Canada; 7 Lawson Health Research Institute, London, ON Canada

**Keywords:** Respiratory syncytial virus, Hospital admission, Temperature, Children

## Abstract

**Background:**

Respiratory syncytial virus (RSV) infection is a major cause of hospitalization in young children in Canada, despite routine immunoprophylaxis in those with medical risk factors. We aimed to determine if cold temperatures are associated with RSV hospitalization.

**Methods:**

We conducted a population-based nested case–control study of children in Ontario, Canada, using health administrative data. We compared children hospitalized for RSV between September 1, 2011 and August 31, 2012 to age and sex matched controls. We used multivariable logistic regression to identify associations between minimum daily temperature and RSV hospitalizations with adjustment for sociodemographic and environmental factors.

**Results:**

We identified 1670 children with RSV hospitalizations during the study period and 6680 matched controls. Warmer temperatures (OR = 0.94, 95%CI: 0.93, 0.95) were associated with lower odds of RSV hospitalization. Southern ecozone (OR = 1.6, 95%CI: 1.2, 2.1), increased ozone concentration (OR = 1.03, 95%CI: 1.01, 1.06) and living in a lower income neighbourhood (OR = 1.3, 95%CI: 1.1, 1.5) significantly increased the odds of RSV hospitalization, as did living in a household with a larger number of siblings in a sub-cohort of children (OR = 1.34, 95%CI: 1.26, 1.41).

**Conclusions:**

In Ontario, the likelihood of having an RSV hospitalization is associated with colder temperature exposures and socioeconomic factors.

## Background

Respiratory Syncytial Virus (RSV) is a major cause of lower respiratory tract infection, accounting for approximately 85% of bronchiolitis and 20% of pneumonia diagnoses in children [1, 2]. Young children are particularly vulnerable to severe RSV infections. In Canada, RSV related disease is responsible for 9% of all hospitalizations in infants, as well as intensive care unit admissions, and even death [3]. Beyond the acute morbidity and mortality associated with the RSV infection itself, there is also evidence of an association between RSV bronchiolitis in infancy and higher risk of recurrent wheeze and asthma [4, 5] as well as sleep disordered breathing in later childhood [6]. Reducing the risk of severe RSV infection might then have downstream benefits on the risk of subsequent respiratory diseases.

Methods to reduce the frequency of severe RSV infection have focused on providing immunoprophylaxis to children with a major risk factor for severe RSV infection (i.e. hospitalization). Prophylaxis consists of administering monthly injections of RSV immune globulin to children less than 2 years of age during November to March when RSV is most prevalent. In Ontario, the specific high-risk criteria that make a child eligible for publicly funded RSV immunoprophylaxis include prematurity, chronic lung disease, congenital heart disease and trisomy 21, with approximately 2% of children meeting these criteria and receiving prophylaxis each year. (See Supplementary Table 1 for Ontario RSV immunoprophylaxis criteria).

Despite efforts to provide immunoprophylaxis to those known to be most at risk, RSV continues to be a major public health concern globally [7, 8]. This concern has prompted the World Health Organization to include RSV infection in children as one of its priorities for disease surveillance and prevention [9]. In Canada, RSV hospitalization rates have remained stable in the past decade [3, 10]. In Ontario, Canada’s most populous province (population 14 million [11]), there are an average of 1500 hospitalizations due to RSV per year and 85% of these children do not have a major risk factor that would have qualified them for immunoprophylaxis [3]. It could be postulated then that factors beyond medical comorbidities might contribute to RSV risk. If elucidated, these factors could be targeted in new strategies or incorporated in risk prediction tools to reduce the burden of severe RSV disease.

Where RSV demonstrates a seasonal infection pattern that varies by population and geography [12], there is some evidence that specific climate factors might be associated with RSV infections [13]. In European countries with temperate climates, for example, RSV infections were seen to be associated with lower air temperatures and higher relative humidity [14, 15]. Such studies have not been conducted in North America. In this study we aimed to determine whether temperature, namely the mean minimum daily temperature during the time of likely exposure to RSV is associated with the frequency of RSV hospitalizations in Ontario, Canada. Secondary objectives were to identify the contribution of additional geographic factors on the likelihood of RSV hospitalization.

## Methods

### Overview

Using linked health administrative databases from Ontario, Canada, we performed a population-based matched nested case-control study of children 0–36 months of age. Ontario’s health administrative data contains longitudinal inpatient and outpatient health care records from 1991 for all legal residents of Ontario with a valid health card (> 99% of the population). We identified all children hospitalized for RSV between September 1, 2011 – August 31, 2012 and compared their mean temperature exposure and other geographic and demographic characteristics to an age- and sex- matched cohort of children who were not hospitalized for RSV. This study was approved by the Research Ethics Board of the Children’s Hospital of Eastern Ontario and all analyses were conducted at ICES. ICES is an independent, non-profit research institute whose legal status under Ontario’s health information privacy law allows it to collect and analyze health care and demographic data, without consent, for health system evaluation and improvement.

### Patients and setting

**Fig. 1:**
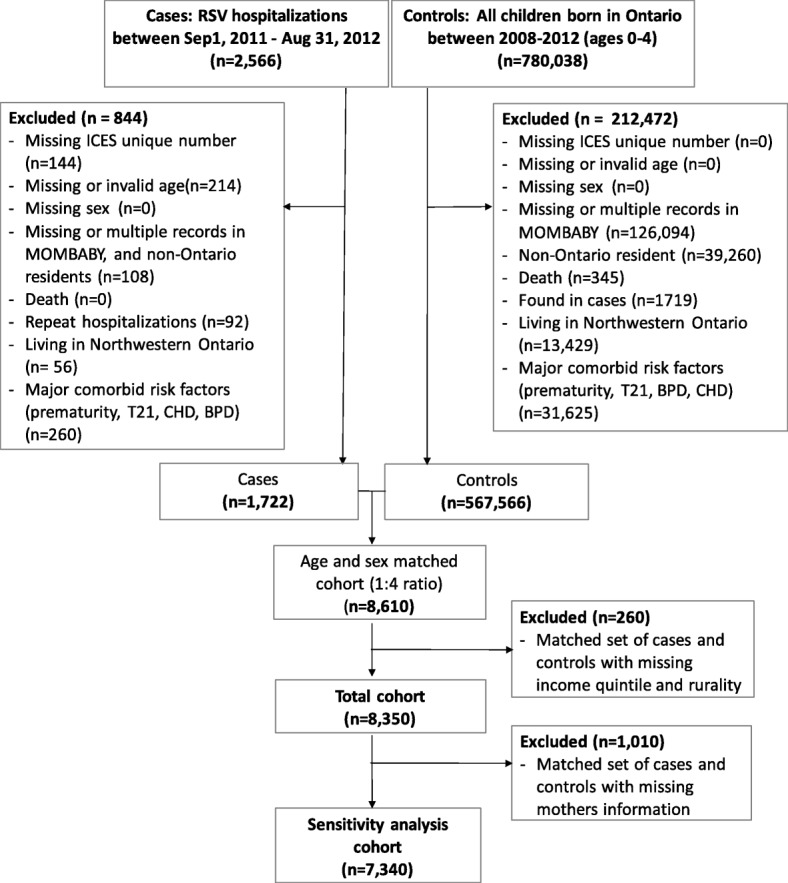
Cohort creation. Controls represent children who were not hospitalized. LHIN = Local Health Integration Network, T21 = trisomy 21, BPD = bronchopulmonary dysplasia, CHD = congenital heart disease

All children living in the province of Ontario and who were between 0 and 36 months of age between Sept 1, 2011 – Aug 31, 2012 were eligible for cohort inclusion. We chose to include only young children as this would capture the majority of RSV hospitalizations [16]. Children who were hospitalized for RSV were matched 1:4 to children who were not hospitalized for RSV over the study period based on age (same year of birth ±1 month) and sex. RSV exposure status, and whether they were RSV positive or negative was not examined for the non-hospitalized children. We excluded children who did not have a valid ICES unique identification number or had missing information on age, sex, or data errors (e.g. death date before birth date). Children were also excluded if we were unable to link their data to the health record of their mother at birth, if they were not residing in Ontario during the study period and if they died on the index date. Furthermore, only the first hospitalization per child was included in analysis (Fig. 1). The province of Ontario is divided into 14 Local Health Integration Networks (LHINs) representing units of health services administration that correspond to geographic areas that would be serviced by specific tertiary care pediatric centres. The majority of children residing in LHIN 14 (representing northwestern Ontario) would usually be hospitalized outside Ontario (in a neighbourhing province); as such we also excluded children residing in this LHIN due to our inability to capture their health services use data. Additionally, as we were not able to obtain data for which children did or did not receive immunoprophylaxis for RSV (i.e. RSV immune globulin), a significant confounder for RSV hospitalization, we excluded children who would typically receive this treatment based on public funding criteria [17]. As such, using previously published diagnostic codes [10] all children who were born prematurely (gestational age < 36 weeks), or had a diagnosis of trisomy 21, bronchopulmonary dysplasia, or congenital heart disease were excluded.

### Data sources

Ontario administrative databases used in this study (see details in Supplementary Table 2) included: the Ontario Health Insurance Plan (OHIP) database which contains physician billing and diagnostic information; the Canadian Institutes for Health Information Discharge Abstract Database (CIHI-DAD) which contains information on hospital admissions; the MOMBABY database which links children to their birth mothers; the Ontario Registered Persons Database (RPDB) which includes information on all births and deaths and enables linkage using postal code information with Canadian census to inform geographic variables at the neighbourhood level; the Ontario Ministry of Natural Resources’ spatial boundary files which define Ontario’s ecological land classifications [18]; Environment and Climate Change Canada’s Canadian Urban and Land Surface External Modelling System data (a.k.a GEMSURF) [19] that provides hourly temperature data at 1 km resolution; and the Air Quality Health Index [20], which was created by Environment and Climate Change Canada in collaboration with Health Canada and provides annual summaries for air quality parameters across Ontario. These datasets were deterministically linked using unique encoded identifiers and analyzed at ICES.

### Outcome

RSV hospitalization was defined using a previously published validated algorithm [10] using ICD-10 diagnosis codes (J12.1, J20.5, J21.0, and B97.4) that has a reported sensitivity of 97.9%, specificity of 99.6%, positive predictive value of 96.9% and negative predictive value of 99.4%. We chose this as the outcome of interest as RSV hospitalization, rather than infection alone, would imply a more ‘severe’ RSV infection with important impact on health, health service utilization and healthcare cost.

### Primary exposure and covariates

Temperature data was summarized daily and by dissemination areas (i.e. small geographic areas composed of one or more neighborhoods with a population of approximately 400–700 persons) [21]. Exposures were assigned to each child based on the date of RSV hospital admission and location of residence. Children who were not hospitalized for RSV (i.e. controls) were assigned the same index date as their matched case. The primary exposure variable was the mean minimum temperature in the 7-day period falling 7–13 days prior to RSV hospitalization, representing the time of exposure to and infection with RSV. We chose this timeframe as it represents the incubation for RSV which typically ranges between 4 and 6 days (and up to 8 days) and period of peak symptoms (and likely time of hospitalization), which develops 3–5 days following incubation [22]. For the non-hospitalized controls, the same exposure period (i.e. 7–13 days prior to hospitalization of the matched RSV hospitalized case) was used for determination of the mean minimum temperature.

Covariates included: the number of live births per mother as a proxy for number of siblings in the home which is a reported risk factor RSV infection [23]; whether the child was living in a rural or urban residence (rural defined as community size < 10,000 persons); distance to the nearest hospital (“as the crow flies”) based on the postal code of the child’s residence compared to hospital location; and the neighbourhood income quintile (1 = lowest). We further included the ecozone of the child’s neighbourhood (North vs South). Ecozones are large geographic areas defined by distinct bedrock geology features that can influence the local climate and ecosystem [24]. Lastly we included annual summaries of air quality health index (AQHI) which represents the relative health risk of exposure to a mixture of common air pollutants including ground level ozone (O3), fine particulate matter (PM_2.5_), and nitrogen dioxide (NO_2_) all measured in parts per billion (ppb); AQHI is reported on a scale of 1–10+ (10+ = very high health risk) [25, 26].

### Analysis

Baseline variables were compared between children hospitalized for an RSV infection and age and sex-matched children who were not hospitalized for RSV over the study period. We used one-way ANOVA or Chi Square tests to compare the means and medians of continuous variables and the proportions of categorical variables, respectively, and we calculated standardized differences. A multivariable conditional logistic regression analysis including non-collinear covariates was conducted to identify significant independent predictors of RSV hospitalization. As information about number of live-births per mother was missing from > 10% of the cohort, we included this variable in a sensitivity analysis, and not in the primary model. All *p*-values < 0.05 were considered statistically significant.

## Results

We identified 1670 children who were hospitalized for RSV infection during the study period and 6680 matched controls (Table 1). The median age of both cases and controls was 4.0 months, (IQR 2.0–13.0 months). There were slightly more males (63.8%) than females who were hospitalized for RSV and the number of births per mother was higher among RSV cases than controls (2.5 ± 1.1 vs 2.2 ± 1.1 *p* < 0.001). The mean minimum daily temperature during the 7–13 days prior to RSV hospitalization was colder among RSV cases than during a similar time-period in the controls (− 4.6°Celsius (C), ± 4.8 °C vs − 2.4 °C ± 6.6 °C, p < 0.001).

**Table 1:** Characteristics of RSV-hospitalized children (cases) versus controls

Characteristics	Cases *N* = 1670	Controls *N* = 6680	Overall *N* = 8350	*P*-value
Age at index date in months [(median, (IQR)]	4.0 (2.0–13.0)	4.0 (2.0–13.0)	4.0 (2.0–13.0)	1
Female [N, (%)]	649 (44.2%)	2596 (44.2%)	3245 (44.2%)	1
Number of live births per mother (mean ± SD)	2.5 ± 1.1	2.2 ± 1.0	2.2 ± 1.0	<.001
Neighbourhood Income quintile [N (%)]				
1 - lowest	351 (23.9%)	1205 (20.5%)	1556 (21.2%)	0.06
2	265 (18.1%)	1160 (19.8%)	1425 (19.4%)	
3	307 (20.9%)	1260 (21.5%)	1567 (21.3%)	
4	315 (21.5%)	1272 (21.7%)	1587 (21.6%)	
5 - highest	230 (15.7%)	975 (16.6%)	1205 (16.4%)	
Rural [N (%)]	158 (10.8%)	552 (9.4%)	710 (9.7%)	0.11
Distance to nearest acute care hospital in km (Mean ± SD)	6.0 ± 6.3	5.9 ± 7.2	5.9 ± 7.0	0.49
Ecozones [N (%)]				
South	1396 (95.1%)	5597 (95.3%)	6993 (95.3%)	0.72
North^a^	72 (4.9%)	275 (4.7%)	347 (4.7%)	
Annual AQHI^b^ (Mean ± SD)	3.2 ± 0.3	3.2 ± 0.3	3.2 ± 0.3	<.001
Yearly mean of daily max NO_2_^c^ (ppb) (Mean ± SD)	19.5 ± 4.7	20.3 ± 4.9	20.1 ± 4.8	<.001
Yearly mean of daily max O_3_ (ppb) (Mean ± SD)	39.9 ± 2.0	39.8 ± 1.8	39.8 ± 1.9	0.07
Yearly mean of daily max PM2.5^c^ (μg/m^3^) (Mean ± SD)	15.2 ± 1.9	15.2 ± 1.7	15.2 ± 1.7	0.37
Mean of minimum daily temperature during 7–13 prior to index date, (°C) (Mean ± SD)	− 4.6 ± 4.8	− 2.4 ± 6.6	−2.8 ± 6.3	<.001

*IQR* Interquartile range, *SD* Standard deviation, *AQHI* Air quality health index, *ppb* Parts per billion, *μg/m*^*3*^ Microgram per cubic metre, ^*o*^*C* Degree Celsius

^a^Combination of North Central/West and Northeast regions

^b^Linked by census dissemination area = approximately 400–700 persons per neighbourhood

^c^Linked by forward sortation area (FSA); Ontario is divided into 513 FSAs

**Fig. 2:**
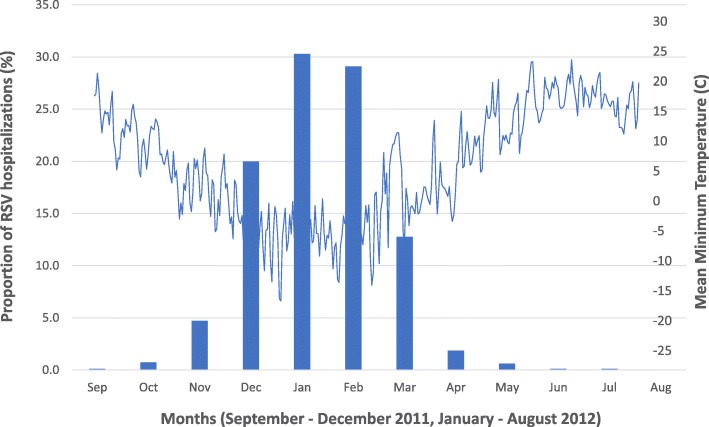
Frequency distribution of RSV hospitalizations by month. *During September 2011, and June – August 2012, the number of hospitalizations was < 6 per month. Exact values are not shown in order to prevent re-identification of patients. Mean minimum temperature represents the lowest daily temperature at each weather station, averaged across the province of Ontario. C = degrees Celsius”

The highest frequency of RSV hospitalizations occurred between the months of November 2011 to March 2012 (Fig. 2). The odds of RSV hospitalization decreased per 1 °C increase in mean daily temperature (OR = 0.94, 95% CI: 0.93, 0.95). In our adjusted analysis, we found that warmer mean daily temperature (OR = 0.94, 95% CI: 0.93, 0.95) was still associated with decreased odds of RSV hospitalization. On the other hand, lower neighbourhood income quintile (OR 1.28, 95% CI: 1.07, 1.54), living in the southern ecozone (OR 1.60, 95% CI: 1.21, 2.13) and higher annual ozone concentrations per 1 ppb increase (OR 1.03, 95% CI: 1.01, 1.07) were associated with increased odds of RSV hospitalization (Table 2).

**Table 2:** Conditional logistic regression for odds of RSV hospitalization among children in Ontario

Unadjusted model	OR	LCL	UCL	*P*-value^4^
Minimum daily temperature pre RSV^1^	**0.94**	**0.93**	**0.95**	**<.0001**
Adjusted model				
Minimum daily temperature pre RSV^1^	**0.94**	**0.93**	**0.95**	**< 0.001**
Neighbourhood Income quintile				
1 - lowest income	**1.28**	**1.07**	**1.54**	**0.006**
2	0.98	0.81	1.18	0.82
3	1.04	0.87	1.25	0.65
4	1	0.84	1.20	0.99
5 - highest income	1	Ref	Ref	Ref
Rural				
Yes	1.11	0.90	1.36	0.34
No	1	Ref	Ref	Ref
Distance to nearest hospital	1	0.99	1.01	0.56
Ecozones				
South	**1.60**	**1.21**	**2.13**	**0.001**
North	1	Ref	Ref	Ref
Annual AQHI^2^	0.85	0.66	1.09	0.19
Annual O3^3^	**1.03**	**1.01**	**1.07**	**0.03**

*RSV* Respiratory syncytial virus, *OR* Odds ratio, *LCL* 95% lower confidence limit, *UCL* 95% upper confidence limit, *Ref* Reference value

^1^Mean minimum (low) daily temperature during 7–13 days period prior to RSV hospitalization (representing time during likely RSV exposure and infection)

^2^AQHI = Air quality health index and ranges from 0 to 10+, higher value indicates worse air quality

^3^O3 = ground level ozone concentration in parts per billion

^4^*p* < 0.05 indicates statistically significant associations

For our sensitivity analysis, we included 1468 children where information about Ontario-born siblings was available. In this analysis, warmer mean daily temperature was still associated with lower odds of RSV hospitalization (OR 0.94, 95% CI: 0.92, 0.95), whereas lower neighbourhood income quintile (OR 1.26, 95% CI: 1.03, 1.53), Southern ecozone (OR 1.44, 95% CI: 1.07, 1.95), and more siblings, as approximated by the number of live births of the mother (OR 1.34, 95% CI: 1.26, 1.41), were associated with increased odds of RSV hospitalization (Table 3).

**Table 3:** Conditional logistic regression for odds of RSV hospitalization among children in Ontario (including covariate: number of siblings) – sensitivity analysis

Adjusted model	OR	LCL	UCL	*P*-value^2^
Minimum daily temperature pre RSV^1^	**0.94**	**0.92**	**0.95**	**< 0.001**
Neighbourhood Income quintile				
1 - lowest income	**1.26**	**1.03**	**1.53**	**0.02**
2	0.98	0.80	1.19	0.81
3	1.03	0.85	1.25	0.78
4	1.02	0.84	1.24	0.83
5 - highest income	1	Ref	Ref	Ref
Rural				
Yes	0.96	0.77	1.20	0.73
No	1	Ref	Ref	Ref
Distance to nearest hospital	1	0.99	1.01	0.45
Ecozones				
1 - South	**1.44**	**1.07**	**1.95**	**0.02**
2 - North	1	Ref	Ref	Ref
Annual AQHI	0.84	0.65	1.10	0.20
Annual O3	1.02	0.98	1.05	0.38
Mothers number of live births	**1.34**	**1.26**	**1.41**	**<.0001**

*RSV* Respiratory syncytial virus, *OR* Odds ratio, *LCL* 95% lower confidence limit, *UCL* 95% upper confidence limit, *Ref* Reference value, *AQHI* Air quality health index (higher value = worse air quality), *O3* Ground level ozone concentration

^1^Mean minimum (low) daily temperature during 7–13 days period prior to RSV hospitalization (representing time during likely RSV exposure and infection)

^2^*P* < 0.05 represents statistical significance

## Discussion

Hospital admissions for respiratory infections due to RSV remain a significant health problem among young children and this is despite provision of immunoprophylaxis to children with medical factors placing them most at risk of this outcome. In an effort to find new strategies to reduce the burden of severe RSV we aimed to identify additional environmental factors that might be associated with RSV hospitalization. In this study we found that colder air temperatures during the most prevalent exposure period for RSV infection are associated with increased likelihood of RSV hospitalization.

This finding is in alignment with other studies of RSV infection in temperate climates that have noted a relationship with colder air temperature. For example, one study from the UK found that an increase in mean daily temperatures in central England observed over 2 decades (1981–2004) (i.e. global warming), was associated with a gradual shortening of the RSV season over time [27]. Our identification of a relationship between temperature and severe RSV infections may reflect RSV - host virulence factors or human behavioural factors influencing transmission.

There is some evidence for increased stability of RSV in liquid droplets at lower temperatures which might lead to prolonged survival of RSV on environmental surfaces and increased infection risk during colder periods [28, 29]. Nasal and airway epithelium may be more susceptible to RSV infection and less able to clear infections when exposed to cold. For example, disruption and damage to the respiratory mucosal barrier caused by dry and cold air conditions can increase risk of penetration and infection by RSV [30]. Furthermore, inhalation of cold air slows the mucociliary escalator (a non-specific respiratory immune defence) and causes increased susceptibility to infection as well as impaired ability to effectively clear infections which may lead to increased severity and hospitalization [23, 28, 29, 31].

However, another explanation for our finding of increased odds of RSV infection with cold temperatures could be due to the tendency of people to spend more time indoors during cold weather periods. In the setting of a household with one infected member, this would increase the duration of exposure of the others to the virus and facilitate RSV infection transmission amongst family members including vulnerable young children. Increased duration of exposure due to indoor cohorting may also increase the inoculum size and viral load. This, in combination with an impaired naso-epithelial barrier and reduced mucociliary clearance if there were any recent cold air contact in the exposed children, would likely increase the severity of illness and risk of subsequent hospitalization [32, 33]. In our sensitivity analysis we did confirm that a higher number of siblings was independently associated with an increased likelihood of having an RSV admission, and previous studies have shown that infants are most likely to contract RSV from household members [34]. A similar mechanism has been hypothesized for the observation of increased RSV infection during periods of increased heat and humidity in tropical countries when people may spend more time indoors. Studies of indoor air quality conducted in Canada’s far northern regions where average household size is typically more than 6 persons further support this mechanism and have shown an association between reduced indoor ventilation and increased rates of respiratory infections, including RSV, among young Inuit children [35].

The link between cold air temperatures and RSV hospitalization in our study was independent of multiple confounding factors that we included in our adjusted model, including geographic variables and socioeconomic status. Indeed we did observe an association between lower neighbourhood income quintile and increased odds of RSV admission as previously noted in the literature [36, 37]. The relationship between respiratory illnesses, and overall greater acute health services use with lower socioeconomic status has been well documented in many Canadian studies. In our primary model, we noted a weak association between ground level ozone and increased likelihood of RSV hospitalization. Associations between increased ambient air pollution levels (e.g. PM 2.5, Ozone) and respiratory tract infections, including bronchiolitis in children have been reported previously in the literature [38–40]. One proposed mechanism for this relationship is that particulate matter and other air pollutants cause airway inflammation and impair mucociliary clearance, thereby reducing lung defenses against infection [41]. Nonetheless, we did not see the association between RSV hospitalization and air pollution in our sensitivity analysis or find any association with overall AQHI. These potential associations may be underestimated in our study as we were only able to capture *annualized* ozone levels and AQHI and it is possible these may not be accurate indicators of the air quality during the time of RSV infection.

Though not the main focus of our study, we did note a relationship between RSV admission and geographical location. Living in the southern ecozones (warmer climates, more urbanization) was associated with an increased odds of RSV admission that was not related to rural versus urban residence. It is possible that environmental parameters beyond temperature or air quality, such as humidity or barometric pressure, as identified in other studies, as well as other unmeasured ecological factors may influence the risk of RSV infection and illness severity among Ontario children.

It has been previously shown that the majority of RSV hospitalizations among children in Ontario occur in those who would not have received immunoprophylaxis [10]. Our work suggests an opportunity to identify risk factors beyond comorbid health conditions that could be targeted to refine our current criteria for providing this preventative therapy. For example, a cost-benefit analysis could be performed to determine the effect of extending criteria for immunoprophylaxis to additional vulnerable populations just during the coldest winter months, rather than for the entire RSV season, or considering socioeconomic factors or number of siblings in the home when determining who qualifies for RSV immunization. In our study we excluded children of < 36 weeks gestational age as they might have qualified for immunoprophylaxis, but many children between gestational ages 32–35 weeks in fact do not receive this intervention as they do not meet all qualifying criteria. For this population in particular, the consideration of additional risk factors in determining who meets criteria for public provision of immunoprophylaxis could have significant health impact and further reduce RSV hospitalizations which have remained constant in the last decade.

The use of health administrative data in this study allowed us to explore the association between climate factors and RSV hospitalization across the entire population of the vast geographic expanse of Ontario. Though this methodology provided significant power, there are some limitations, including lack of information on potential contributory factors such as environmental tobacco smoke or indoor air quality. In an effort to eliminate the confounding influence of immunoprophylaxis on the odds of RSV admission, we excluded all children who would have possibly met the most common criteria for immunoprophylaxis for RSV such as prematurity, or the presence of chronic lung disease, congenital heart disease or trisomy 21. As such, our study results are not generalizable to all children. It is also possible that some children in our cohort of RSV cases may have received immunoprophylaxis (due to individual risk factors identified on a case-by case basis), though we would expect this to be a very small proportion and unlikely to alter the main study findings. Finally, other spatio-temporally clustered factors beyond the geographic and climate variables explored in this study may account for some of the associations seen, but given our data limitations, these were not examined here.

## Conclusions

Respiratory syncytial virus infection is common in children and the majority of hospitalizations occur in children without traditional risk factors for severe infection or indications for publicly funded immunoprophylaxis. In this study we demonstrated that RSV hospitalizations follow a seasonal distribution and are associated with colder temperatures in the time leading up to hospitalization, during the period of likely virus exposure. This study provides information on environmental and household risk factors that are not currently targeted for reduction of RSV morbidity but may be promising areas for further study and future preventative strategies.

## Supplementary information

**Additional file 1:** **Table S1.** Criteria for publicly funded RSV prophylaxis in Ontario.

**Additional file 2:** **Table S2.** Health administrative databases and study variables.

## Data Availability

The data set from this study is held securely in coded form at ICES. While data sharing agreements prohibit ICES from making the data set publicly available, access may be granted to those who meet pre-specified criteria for confidential access, available at www.ices.on.ca/DAS*.* The full data set creation plan and underlying analytic code are available from the authors upon request, understanding that the programs may rely upon coding templates or macros that are unique to ICES.

## Abbreviations

ANOVA: Analysis of variance
AQHI: Air quality health index
CIHI: Canadian Institute for Health Information
CIHI-DAD: Canadian Institutes for Health Information Discharge Abstract Database
GEMSURF: Environment and Climate Change Canada’s Canadian Urban and Land Surface External Modelling System data
ICD-10: International Classification of Disease
ICES: Institute for Clinical Evaluative Sciences
IQR: Interquartile range
LHIN: Local Health Integration Network
MOHLTC: Ontario Ministry of Health and Long-Term Care
NO_2_: Nitrogen dioxide
O3: Ozone
OHIP: Ontario Health Insurance Plan
PM_2.5_: Fine particulate matter
ppb: Parts per billion
REB: Research Ethics Board
RPDB: Ontario Registered Persons Database
RSV: Respiratory syncytial virus
