# Supplementary material for: The association between climate, geography and respiratory syncitial virus hospitalizations among children in Ontario, Canada: a population-based study
Source: BMC Infect Dis. 2020 Feb 19;20:157. doi: 10.1186/s12879-020-4882-6 (PMC7031991; doi:10.1186/s12879-020-4882-6)
Supplement: Supplementary file 1 — Additional file 1: Table S1. Criteria for publicly funded RSV prophylaxis in Ontario. [file 12879_2020_4882_MOESM1_ESM.docx]

**Supplementary Table 1. Criteria for publicly funded RSV prophylaxis in Ontario**

| Infants born prematurely at ≤ 32 completed weeks gestation and aged ≤ 6 months at the start of, or during, the local RSV season; or |
| --- |
| Infants 33 – 35 completed weeks gestation and aged ≤ 6 months at the start of, or during the local RSV season, who DO NOT live in isolated communities AND have a Risk Assessment Tool Score of 49 to 100; or |
| Infants 33 – 35 completed weeks gestation and aged ≤ 6 months at the start of, or during the local RSV season, and who LIVE IN isolated communities where paediatric hospital care is not readily accessible and ambulance transportation for hospital admission is required; or |
| Children < 24 months of age with Down Syndrome / Trisomy 21; or |
| Children < 24 months of age with bronchopulmonary dysplasia/chronic lung disease (BPD/CLD) and who required oxygen and/or medical therapy within the 6 months preceding the RSV season; or |
| Children < 12 months of age with hemodynamically significant (HS) cyanotic or acyanotic congenital heart disease (CHD); requiring corrective surgery or are on cardiac medication for hemodynamic significant disease. Children 12 – 24 months of age with ongoing HS CHD will be considered on a case-by-case basis. |
| Infants born prematurely at ≤ 32 completed weeks gestation and aged ≤ 6 months at the start of, or during, the local RSV season; or |
| Infants 33 – 35 completed weeks gestation and aged ≤ 6 months at the start of, or during the local RSV season, who DO NOT live in isolated communities AND have a Risk Assessment Tool Score of 49 to 100; or |

Reproduced from Ontario Ministry of Health and Long-term Care^1^

**Reference:**

1. Respiratory Syncytial Virus Prophylaxis for High-Risk Infants Program. 2019. (Accessed 2019-02-20, at <http://www.health.gov.on.ca/en/pro/programs/drugs/funded_drug/fund_respiratory.aspx>.)
