# Supplementary material for: The association between climate, geography and respiratory syncitial virus hospitalizations among children in Ontario, Canada: a population-based study
Source: BMC Infect Dis. 2020 Feb 19;20:157. doi: 10.1186/s12879-020-4882-6 (PMC7031991; doi:10.1186/s12879-020-4882-6)
Supplement: Supplementary file 2 — Additional file 2: Table S2. Health administrative databases and study variables. [file 12879_2020_4882_MOESM2_ESM.docx]

**Supplementary Table 2. Health administrative databases and study variables**

| **Name of Database** | **Data Sources** | **Variables** | **Details of use and relevant ICD-10 diagnostic codes** |
| --- | --- | --- | --- |
| CIHI-DAD  (Canadian Institutes of Health Information Discharge Abstract Database) | ICES | -Hospitalization date for RSV*  -Comorbid diagnoses** | *Trisomy 21:*  Q90.0, Q90.1, Q90.2, Q90.9  *Congenital heart disease :* Q20.0-Q20.9, Q21.0-Q21.9, Q22.0-Q22.9, Q23.0-Q23.9, Q24.0-Q24.9, Q25.0-Q25.9, Q26.0-Q26.9  *Bronchopulmonary dysplasia :* P27.1, P27.8, P27.9 |
| OHIP  (Ontario Health Insurance Plan) | ICES | Non-Ontario Residents (i.e. no valid OHIP number) | -Exclusion criteria  -encrypted OHIP number used as linking variable |
| RPDB  (Registered Persons Database) | ICES | -Sex  -Age  -Death Date | -Exclusion Criteria |
| POP (population database)  CENSUS  PCCF (Postal Code Conversion Files) | ICES | -Income quintile (1 = lowest)  -Rural residence (<10,000 persons community size) | These databases listed were linked using postal code information to inform these variables |
| LHIN (Local Health Integration Network) | ICES | -Residence in LHIN 14 | -Exclusion Criteria (as many hospitalizations for this group may not be captured) |
| MOMBABY (linked database of mothers and babies born in Ontario) | ICES | -Gestational age  -number of live births per mother (as a proxy for number of children in the home) |  |
| Eco Zones | Ontario Ministry of Natural Resources | North = Northwest (Ontario Shield) + Northeast (Hudson Bay Lowlands)  vs South (Mixedwood Plains) | -Geographic ecozone of residence neighbourhood (i.e. postal code falling within an ecozone geographic area) |
| AQHI (Air Quality Health Index) | Environment and Climate Change Canada/Health Canada | -Annual AQHI on Scale ranging from 1 (low risk) to 10 (high risk)  -Annual mean of daily maximum pollutants such as O_3_ (ozone), NO_2_ (nitrogen dioxide), and PM_2.5_ (particulate matter) | Annual AQHI and single pollutant measures are by areas (i.e. census division or forward sortation area) |
| GEMSURF Weather data | Environment and Climate Change Canada | -Minimum daily temperature | Data is collected on an hourly basis at the 1 km resolution in Ontario and summarized daily by dissemination areas |

*RSV = Respiratory Syncitial Virus

**diagnoses are based on previously published ICD-10 codes^1^

**Reference:**

1. Pisesky A, Benchimol EI, Wong CA, et al. Incidence of Hospitalization for Respiratory Syncytial Virus Infection amongst Children in Ontario, Canada: A Population-Based Study Using Validated Health Administrative Data. PLoS One 2016;11:e0150416.
